# An introduction to the methodological corpus of systematic observation and its quality assessment

**DOI:** 10.3758/s13428-026-03189-5

**Published:** 2026-10-13

**Authors:** Daniel López-Arenas, Susana Sanduvete-Chaves, Salvador Chacón-Moscoso

**Affiliations:** 1 Facultad de Psicología, Departamento de Psicología Experimental, Universidad de Sevilla, Campus Ramón y Cajal, C/Camilo José Cela S/N, 41018 Seville, Spain; 2 Universidad Autónoma de Chile, Santiago, Chile

**Keywords:** Observational methodology, Mixed methods, Behavioral stream, Methodological quality, Tutorial

## Abstract

Observational methodology (OM) has evolved into a rigorous and flexible mixed-methods framework for studying spontaneous behavior in natural settings. By systematically recording behavioral streams and converting qualitative data into quantitative parameters, OM provides ecologically valid evidence often unattainable in experimental designs. This article presents a comprehensive methodological framework that synthesizes the conceptual, methodological, and analytical foundations of OM, along with recent advances in quality assessment. It traces OM’s evolution from qualitative traditions to a mature scientific methodology grounded in technological innovations and robust analytical procedures. A central element is the taxonomy of observational designs, which guides decisions on instrument development, sampling, and data analysis. This paper examines the structure of observational instruments, highlighting the complementary roles of structured category systems and flexible field formats, as well as the growing use of hybrid instruments that combine theoretical rigor with practical flexibility. Key components of the QUAL-QUANT-QUAL sequence are described, including behavioral segmentation, qualitative data typologies, and their transformation into quantitative parameters such as frequency, order, and duration. Analytical techniques used in OM include classical statistics and three field-specific procedures: lag sequential analysis, polar coordinate analysis, and T-pattern detection, which together enable precise identification of recurrent behavioral patterns. The article also addresses quality assessment, reviewing key frameworks that promote transparency, standardization, and evidence synthesis across behavioral science domains. Overall, this work offers an updated reference for designing, implementing, evaluating, and synthesizing studies based on OM.

## Introduction

Observational methodology (OM) is based on the systematic recording of spontaneous behavior in natural settings, as well as on rigorous quality control of the resulting data, and the integration of qualitative and quantitative components through advanced quantitative techniques. This research methodology is regarded as one of the most effective approaches available to the behavioral sciences for developing studies with high ecological validity (Anguera, 1990). In the domains of psychology and social sciences, naturalistic intervention is a complex process that differs from actions such as manipulating variables or forming experimental groups, which are artificial for participants. In recent years, OM has undergone significant advances due to technological developments (Morales-Sánchez et al., 2020; Wu et al., 2021). This approach involves minimal intervention, providing information less affected by extraneous variables such as social desirability bias or researcher-related influences (Anguera et al., 2018b). Additionally, this methodology is independent of standardized measurement instruments (Portell et al., 2015), allowing OM-based studies to be conducted in diverse contexts, including sports fields (Maneiro et al., 2022) and zoos (Kohut & Katona, 2022), for example. OM can be regarded as both flexible and rigorous. It follows the fundamental stages of hypothesis formulation, data collection and analysis, and interpretation of results. This process enables the systematic study of everyday behaviors and interactions that are normally inaccessible within experimental designs (Anguera, 2010).

Conventionally, observation was associated with limited systematization and with researchers’ reliance on subjective narratives characteristic of ethnographic studies. Since its inception in the 1970 s, observation was not originally structured as a methodology but rather classified within qualitative approaches. This is because it tended to capture reality narratively as it unfolded, was recorded manually, and was not recognized as a scientific research method (Anguera, 1979). However, since the 1990 s, OM has undergone progressive refinement within scientific principles, establishing solid methodological foundations in terms of observational design (Anguera et al., 2011) and the systematization of recording and coding (Anguera & Blanco-Villaseñor, 2005).

Today, OM is recognized as a scientific approach characterized by both flexibility and rigor. The flexibility of this methodology has been further enhanced by the development of indirect observation, which complements traditional direct observation based on audiovisual recording and data analysis in fully perceptible observational contexts. Indirect observation refers to analyzing textual materials obtained either indirectly, through transcriptions of verbal behavior in natural contexts such as conversations or debates, or directly, through written materials such as letters, social media messages, or other documents (Anguera et al., 2018b).

In the social and health sciences, the term *observational* is often used as a synonym for *correlational* (Bakeman & Quera, 2011). However, it is essential to distinguish studies based on OM from those classified as *observational studies*, a category prevalent in health research (Poppe et al., 2025). The latter are quantitative designs—such as cohort, case–control, and cross‑sectional studies—that compare empirical groups to identify cause‑and‑effect relationships in contexts where randomization or experimental control is limited (Cochran & Chambers, 1965). In contrast, OM comprises an initial stage of systematic observation, a second stage of quantitative data analysis, and a final stage involving results interpretation. Consequently, OM is regarded as a mixed‑methods approach (Anguera et al., 2017) because it is founded on the transformation and integration of both qualitative and quantitative components (Cresswell & Plano-Clark, 2017).

In this sense, designs based on OM are organized in a QUAL–QUANT–QUAL sequence. The initial qualitative phase begins with defining the observational design. This design then guides the development or selection of an observational instrument for behavior categorization. Subsequently, behavior is systematically recorded and coded using appropriate recording tools to obtain a matrix of qualitative codes. The quantitative phase starts with quality control procedures applied to the recorded qualitative data, followed by the application of quantitative techniques. The matrix of qualitative data is then transformed into quantitative parameters through quantitizing (Chacón-Moscoso et al., 2023; Tashakkori & Teddlie, 1998). These parameters are subsequently analyzed using classical descriptive and inferential techniques, as well as regularity detection procedures characteristic of OM, such as lag sequential analysis, polar coordinate analysis, and T‑pattern detection. The final qualitative phase involves the interpretation of results in light of the initial research question and relevant literature (Anguera et al., 2020). From a mixed-methods perspective, one critique is that the sole source of information in OM is systematic recording. However, recent developments show an increasing integration of data from direct and indirect observation with other information sources, such as categorical or physiological data, among others (Anguera & Hernández-Mendo, 2016).

This article examines the taxonomy of observational designs and the structure of observational instruments, highlighting the complementary roles of category systems and field formats, as well as the growing use of hybrid instruments that combine theoretical rigor with practical flexibility. It also addresses sampling strategies, the correspondence between qualitative data, quantitative parameters, and observational design type, procedures for quality control, analytical techniques, and approaches to methodological quality assessment.

## Observational design

Design plays a crucial role in the research process, given its significant impact on the subsequent implementation, analysis, and dissemination of results (Deventer, 2009). In this regard, one of the most relevant innovations in the methodological framework of systematic observation is observational design, which provides a framework for all situations that may arise in a study using OM (Anguera et al., 2001). Consequently, observational designs form a taxonomy of eight types that guide decisions about the observational instrument, data collection, the type of qualitative data recorded, the type of quantitative parameter into which it is transformed, and the type of data analysis (Anguera, 2003).

The configuration of observational designs is defined by the units of study, temporality, and dimensionality (Anguera et al., 2001). The units of study specify whether behavior is examined at the level of single participants, dyads, or larger groups. This criterion leads to designs that are a) idiographic, in which only one unit is involved (a participant, a dyad, or several participants that constitute a unit), or b) nomothetic, in which multiple units are of interest for independent study. Temporality refers to the classic distinction between cross-sectional and follow-up studies (León & Montero, 2020). In OM, this criterion gives rise to designs that are either a) punctual, which examine a single observational moment without considering change over time, or b) follow-up, which examine the same observational phenomenon across multiple time points. Dimensionality refers to the number of conceptual axes around which spontaneous behavior is structured, regardless of the richness or diversity of response levels recorded. This criterion gives rise to a) unidimensional designs, in which behavioral streams are organized around a single conceptual axis, and b) multidimensional designs, in which behavioral streams are organized around concurrent conceptual axes (Anguera et al., 2001). By combining the criteria of units of study, temporality, and dimensionality, researchers obtain a taxonomy of eight observational designs (Table 1).

**Table 1:** Taxonomy of observational design

Criterion 1 Study Units	Criterion 2 Temporality	Criterion 3 Dimensionality	Observational designs
Idiographic	Punctual	Unidimensional	I/P/U
		Multidimensional	I/P/M
	Follow-up	Unidimensional	I/F/U
		Multidimensional	I/F/M
Nomothetic	Punctual	Unidimensional	N/P/U
		Multidimensional	N/P/M
	Follow-up	Unidimensional	N/F/U
		Multidimensional	N/F/M

*I* Idiographic; *N* Nomothetic; *P* Punctual; *F* Follow-up; *U* Unidimensional; *M*: Multidimensional

**Fig. 1:**
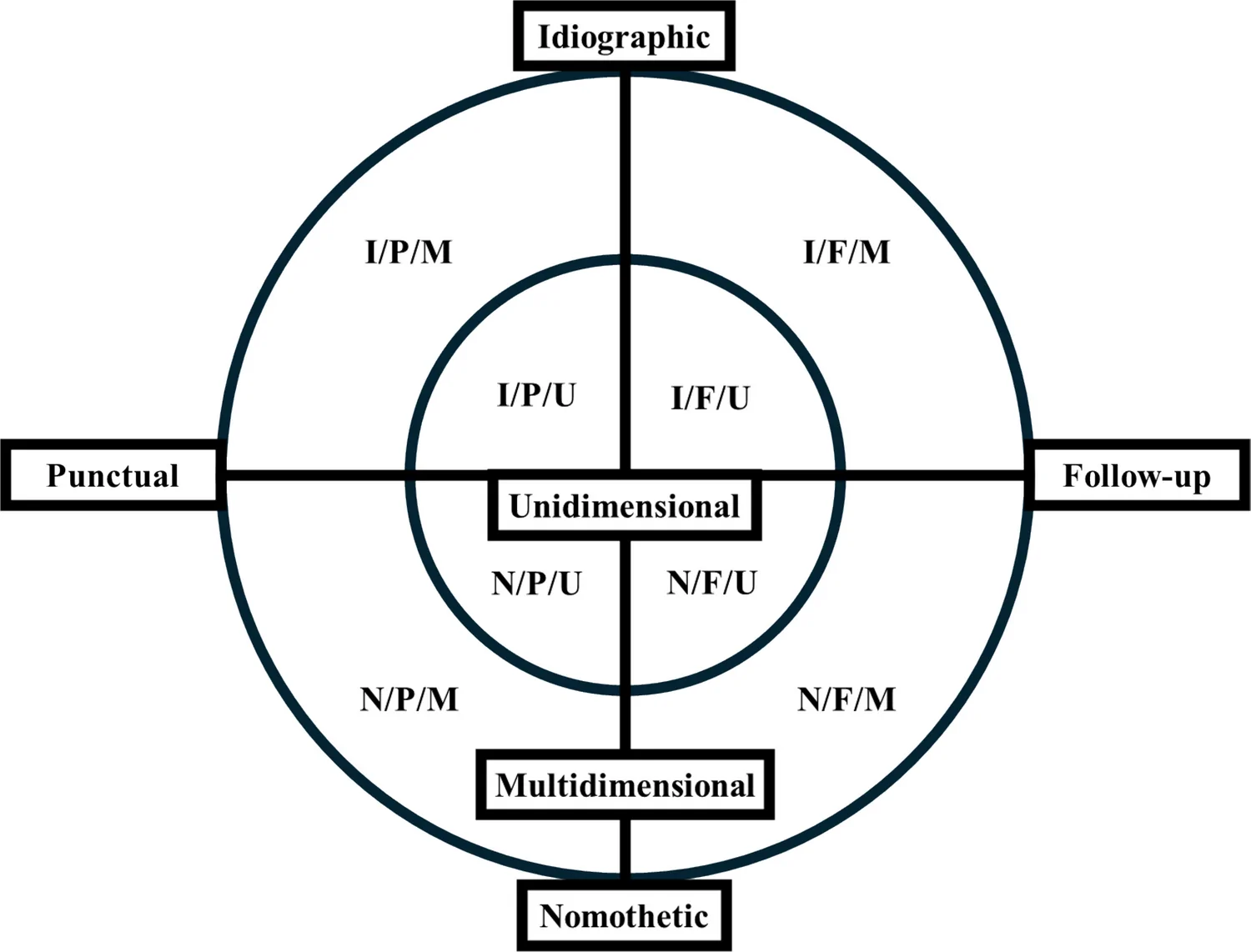
Classification of observational designs by three criteria. *Note*. I: Idiographic. N: Nomothetic. P: Punctual. F: Follow-up. U: Unidimensional. M: Multidimensional

Figure 1 presents the observational designs relying on three criteria.

## Observational instrument

The OM provides a framework for measuring behavior, understood as assigning numbers or labels to discrete behavioral occurrences (Walford et al., 2010). The wide variety of settings in which systematic observation is used makes it impossible to rely only on standardized instruments. Consequently, researchers must devote time to developing an observational instrument for each study, either by creating it from scratch or by selecting and adapting existing ad hoc instruments (Anguera, 2003).

In the context of observation, behavioral streams are continuous, so their analysis requires segmentation into successive units of observation (Anguera et al., 2020). Segmentation can vary in its level of granularity, ranging from molecular (highly detailed segmentation) to molar (relatively broad segmentation). The appropriate level of granularity depends on the research question being addressed (Bakeman & Quera, 2011). Consequently, the researcher must determine the appropriate level of detail, whether more molar or more molecular, for the segmentation process. The two extremes of this continuum have distinct advantages and disadvantages (Anguera et al., 2007), although molecular segmentation is often recommended because it allows units to be subsequently collapsed into more molar ones. Conversely, the reverse process is unfeasible, as distinctions not explicitly identified during segmentation cannot be recovered (Suomi, 1979). Segmentation will be adequate to the extent that the units are a) identifiable in themselves, b) distinguishable from each other, and c) operationally defined so that they are recognizable during the observation session (Anguera & Izquierdo, 2006).

In OM, the term “instrument” is used to denote two distinct entities that serve clearly different functions. The observational instrument is a conceptual coding scheme linked to the content of the study, as it is tailored to the observation context and requires the expertise of the observer. The recording instrument is associated with form, as the observer adopts a position regarding the format in which the behavioral stream is recorded (Anguera et al., 2018a). The fundamental observational instruments are the category system and the field format. However, the combination of both has gradually become the primary structure for the observational instrument. Rating scales are still in use today, but they play a secondary role (Anguera, 1995).

The category system is defined as a coding scheme shaped by both empirical evidence and a theoretical framework, to which the recorded behaviors are assigned. Building a category system begins with compiling an exhaustive list of characteristics of the observational phenomenon. These characteristics are then grouped into conceptually coherent sets based on theoretical considerations. The goal is to obtain a set of categories that is both exhaustive and mutually exclusive, so that any spontaneous behavior in the observation context can be assigned to one—and only one—category within each dimension (Anguera, 2003).

Additionally, the field format is defined as an open list of behaviors spontaneously exhibited in the natural context that have been recorded in earlier stages of exploratory (non‑systematic) observation. Each behavior is assigned a decimal code, which allows hierarchical classification at more molar or more molecular levels of detail as needed (Sánchez-Algarra & Anguera, 2013).

Finally, rating scales have been shown to be useful for assessing social dimensions. Their use is often more efficient than applying codes, particularly when the entities being rated are more molar than the entities being coded (Bakeman & Quera, 2011). While the use of these scales involves a certain degree of subjectivity in the evaluation process as the observer is required to judge how strongly the dimension is expressed (Sánchez-Algarra & Anguera, 2013). This characteristic does not pertain to rating scales in and of itself. The extent to which this is the case is contingent upon the manner in which the rated dimension is operationalized, encompassing the definition of observable indicators and the specification of rating levels. In this sense, rubrics are designed to reduce subjectivity by providing explicit definitions of evaluation criteria and performance levels, thereby promoting greater consistency among raters (Jonsson & Svingby, 2007).

In recent decades, there has been a growing preference for integrating category systems with field formats (Anguera et al., 2007). This combination is feasible when there is a theoretical framework for at least one dimension of the field format and when the focus is on a relatively stable phenomenon rather than a process (Anguera et al., 2018b). The integration of the category system with the field format is based on defining macro‑dimensions, dimensions, and/or sub‑dimensions, depending on the desired level of specificity. For each of these dimensions, a system of categories is nested that meets the criteria of exhaustiveness and mutual exclusivity. Alternatively, there may be an open catalog of mutually exclusive behaviors that does not require a theoretical framework or exhaustiveness (Anguera et al., 2020). This combination gives the observational instrument a balance between theoretical rigor and flexibility and helps overcome the main weaknesses of each type of instrument on its own: the inflexibility of the category system for dynamic and multidimensional processes, and the lack of theoretical grounding in the field format (Anguera et al., 2017). In general, studies that use unidimensional designs rely on category systems and rating scales, whereas those that use multidimensional designs employ field formats or a combination of category systems and field formats (Anguera et al., 2018b).

Because the response levels that make up an observational instrument can be defined from scratch through non‑systematic observation or derived from prior evidence (Bakeman & Quera, 2011), it has become increasingly common to treat ad hoc observational instruments as standardized instruments. This trend is evident in scientific articles that focus exclusively on describing their construction (e.g., Belza, 2015; Blanco-Villaseñor et al., 2000; Caicedo & Calderón, 2020; Castellano et al., 2000; Fabra et al., 2018; Hernández-Mendo & Anguera, 2001; Sainz-de-Baranda, 2002) and in the widespread use of names, acronyms, or abbreviations to refer to them (e.g., Football Observation System or SOF, Anguera et al., 2000; Observational Instrument for the Assessment of Offensive Tactical Actions in Football or VATOF, Caicedo & Calderón, 2020; System for Observing Game Actions in Football or SOCCAF, Castellano, 2000; Multidimensional Motivational Climate Observation System or MMCOS, Fabra et al., 2018; Performance Analysis Offensive in Football or REOFUT, González-Rodenas, 2013; Offensive Process in Football or POF, Lago-Peñas & Anguera, 2003). Consequently, there is a clear trend away from constructing observational instruments entirely from scratch—a lengthy and iterative process—and toward building them by assembling components from existing observational instruments.

Alongside the construction of the observational instrument, the coding manual is drafted. The manual is divided into two sections. First, each code is assigned a name and defined, with illustrative examples provided to facilitate comprehension. These definitions should be concise and include nuances that clarify similarities and differences with other codes with which it might be confused (Bakeman & Quera, 2011). The second section specifies the syntax rules that govern how codes can be combined, and which sequences can result from these combinations (Anguera & Izquierdo, 2006). Regardless of its structure or wording, the coding manual is a fundamental component of OM and deserves particular attention. Consequently, scientific publications based on OM, including reports and journal articles, should provide access to the coding manual (Bakeman & Quera, 2011). Technological advances and the rise of open science over the past decade have made it easier to share coding manuals in different ways, including embedding them in the main text (e.g., Alcover et al., 2019), adding them as supplementary material (e.g., Ortega-Toro et al., 2019), or hosting them in online repositories (e.g., Rodríguez-Morejón et al., 2018).

## Observational sampling

OM is characterized by the need to carry out three distinct sampling processes, because it is necessary to define not only the participants but also the observation sessions and the observational phenomenon within those sessions. Spelling out these sampling decisions substantially strengthens the research design and is essential for adapting the specific steps of OM to the object of study. One of the main requirements is inter- and intra-session consistency, since differences between observation sessions, or within the same session, can compromise the rigor of the study (Anguera, 2003).

Observational sampling is based on the idea that observation sessions must be representative of both the participants included and the behaviors under study. As with other research methods, participant sampling can be clearly defined by inclusion and exclusion criteria. However, given the low level of intervention that characterizes research contexts employing OM, participant sampling is usually done for convenience (Anguera et al., 2018a, b). In other cases, it may be subject to inter- or intra-session sampling (e.g., Ferioli et al., 2023; Pérez-Chao et al., 2023). As a result, it is very likely that all individuals present in the research context at the time of the observation session will initially be considered potential participants, and that later exclusions will be due to failure to meet the inclusion or exclusion criteria. These exclusions are not related to participant sampling itself, but to session or observation sampling (for example, inadequate sessions or a high percentage of unobservability).

Inter‑session sampling is defined as the process of delineating the boundaries of observation sessions (Anguera et al., 2018a). These sessions are understood as sequences of behavioral events that are treated as continuous, despite the presence of planned or unplanned interruptions (Bakeman & Quera, 2011). The criterion used to delimit sessions can be behavioral (a specific behavior must start or stop for the session to begin or end), temporal (a set amount of time must elapse for the beginning or end of the session to be declared), or mixed (a combination of behaviors and time intervals).

Intra‑session sampling, in contrast, takes place during the recording conducted within each observation session. Sampling can therefore be either continuous or discontinuous. Continuous sampling, also known as transition‑triggered recording or RAT, involves recording data continuously as it occurs, thereby ensuring the preservation of the complete chronological sequence and timing of behavioral transitions. Discontinuous sampling, also known as time‑triggered recording or RAUT, involves recording data at predetermined time points. In RAUT, the observation session is divided into equal time intervals, and three main techniques can be used: a) point sampling, in which the presence of the behavior at a specific moment is recorded; b) partial‑interval sampling, in which the behavior is marked if it occurs at any time during the interval, with additional occurrences ignored; and c) whole‑interval sampling, in which the behavior is recorded only if it occurs throughout the entire interval. RAT and RAUT should be distinguished from simple event recording, a minimal form of continuous sampling that retains neither the transition information characteristic of RAT nor the interval structure of RAUT, registering only the total number of occurrences of a behavior within a session, without preserving sequential or temporal information. Although continuous recording is more demanding logistically, it is generally recommended because, after quantification, it allows estimation of all three quantitative parameters. By contrast, in discontinuous records, estimates of occurrence and duration are limited: partial‑interval recording tends to overestimate, whereas whole‑interval recording tends to underestimate. Finally, simple event recording allows only estimation of frequency parameters (Anguera et al., 2018a, b).

In recent years, there has been a marked increase in the development of recording tools specifically designed for OM. In addition to general‑purpose software such as Microsoft Excel, which can be used for manual spreadsheet‑based recording, there are now freely available programs created to record, play back, and analyze data obtained through systematic observation (Anguera et al., 2017). Prominent examples include LINCE (Gabin et al., 2012; http://observesport.com), LINCE PLUS (Soto et al., 2019; https://lince-plus.com), HOISAN (Hernández-Mendo et al., 2012; http://www.menpas.com), Match Vision Studio (Perea et al., 2006), MOTS (Castellano et al., 2008; http://www.menpas.com), SoccerEye (Barreira et al., 2014), SDIS-GSEG (Bakeman & Quera, 2011), THEME (PatternVision, 2002), and Transana (Wisconsin Center for Education Research [WCER], 2011; https://www.transana.com), among others. Additionally, there are numerous specific indirect observation software programs designed to facilitate the process of recording verbal behavior displayed in conversations or written texts. For instance, the CAQDAS family includes software such as AQUAD6, ATLAS.ti, MAXQDA2, NUDIST, and NVivo, among others, which facilitate the segmentation and coding of texts. Additionally, there are also free programs such as T-LAB (http://tlab.it/en/presentation.php), IRAMUTEQ (www.iramuteq.org), and the software developed by the GIAT group at the University of Padua (www.giat.org).

## Qualitative data

**Fig. 2:**
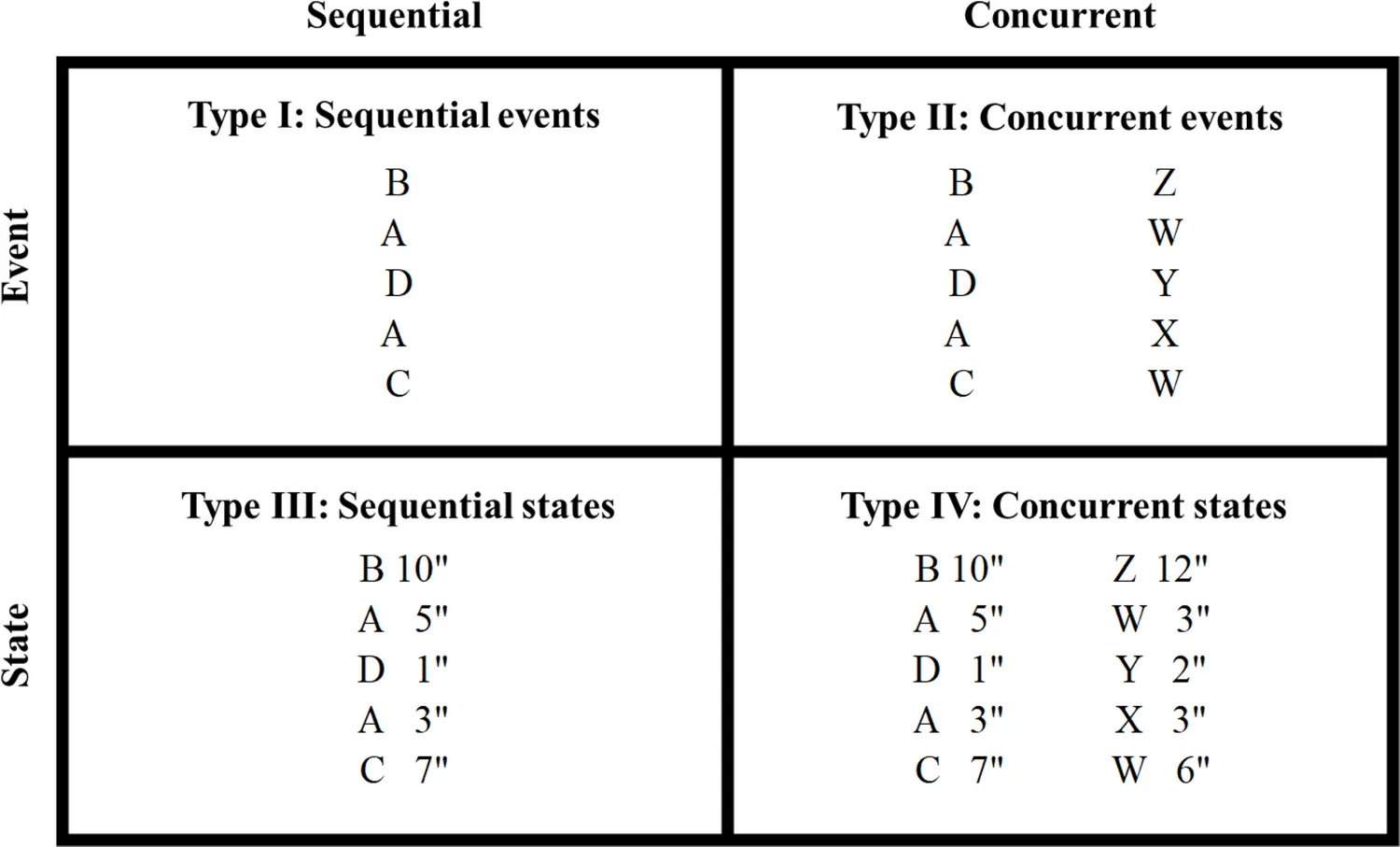
Typology of qualitative data

The qualitative data recorded can be classified into four types: a) type I, a sequence of events within a single behavioral stream; b) type II, a sequence of concurrent events across multiple behavioral streams; c) type III, a sequence of states within a single behavioral stream extending over time; and d) type IV, a sequence of concurrent states across multiple behavioral streams extending over time (Bakeman & Quera, 2011). The data are recorded in a code‑matrix format, where the rows represent the sequence of events or states (time) and the columns represent each dimension of the record. Each row of the matrix contains the codes corresponding to the co‑occurring elements that make up each behavioral unit (Anguera et al., 2020). Figure 2 presents the typology of qualitative data that can be recorded in OM.

Type IV data, which has greater informational potential, contrasts with type I data, which contains less information. A key feature that gives OM its flexibility is the ability to down‑code the typology of qualitative data to lower hierarchical levels. Thus, a record of concurrent states (type IV) can drop all but one behavioral stream and be treated as a record of states in a single stream (type III); it can ignore the temporal units and be treated as concurrent events (type II); and it can drop both properties and be treated as a record of sequential events (type I). Records of sequential states (type III) and concurrent events (type II) can only be reduced to records of sequential events (type I); type III records do so by discarding the temporal‑unit information, and type II records by retaining only one behavioral stream (Bakeman & Quera, 2011).

**Fig. 3:**
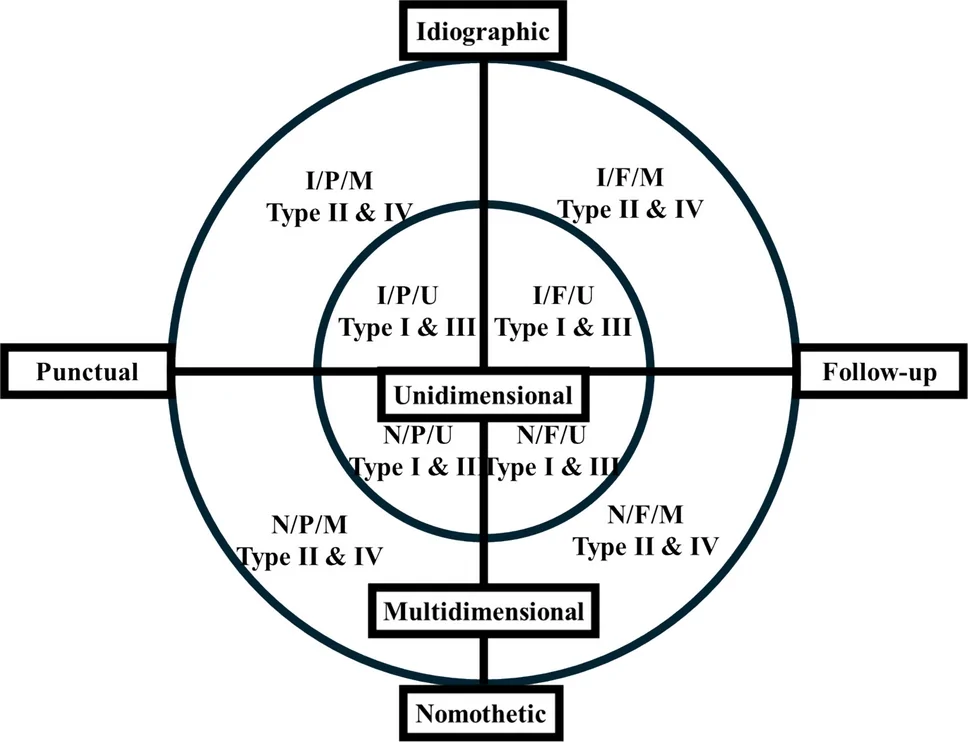
Correspondence between the type of observational design and qualitative data. *Note*. I: Idiographic. N: Nomothetic. P: Punctual. F: Follow-up. U: Unidimensional. M: Multidimensional

In addition, dimensionality, the third criterion that underpins the taxonomy of observational designs, defines the correspondence between each observational design and the type of qualitative data recorded. Consequently, unidimensional designs—whether idiographic or nomothetic, punctual or follow‑up—yield type I data when events are recorded, or type III data when states are recorded, because the focus on a single behavioral stream precludes concurrent data. Conversely, multidimensional designs yield type II data when events are recorded and type IV data when states are recorded (Anguera, 2003). Figure 3 presents the correspondence between the type of observational design and the type of qualitative data.

## Data quality control

It is important to note that observation sessions are subject to sampling error, because they capture only a subset of the spontaneous behavioral stream that occurs in natural contexts (Anguera et al., 2018a). Consequently, it is essential to subject the matrix of recorded codes to rigorous data quality checks, in order to filter the data and give the researcher reasonable assurance about the integrity and reliability of the quantitative parameters that will be analyzed (Blanco-Villaseñor, 1993).

First, in a QUAL–QUANT–QUAL logic, the observer consensus may be understood as a procedure for refining and adjusting the qualitative record prior to quantitative analysis. Then, during quantitizing, data quality control can be examined through inter‑observer reliability (agreement between parallel records made independently by two observers) and intra‑observer reliability (agreement between records made by the same observer after a reasonable interval) (Blanco-Villaseñor, 1989). It is imperative to emphasize that the observational material must remain constant when conducting data quality control. This ensures that any variability observed can be attributed to the coding process rather than to other factors. When it is assumed that an observational measure may be influenced by extraneous factors such as the session, the observer, the recording method, or the recording instrument, generalizability theory offers a framework for integrating these sources of variation within a single structure (Chacón-Moscoso et al., 2006; Cronbach et al., 1972).

The software used to assess inter‑ and intra‑observer reliability, as well as other reliability indices derived from classical psychometric theory, is the same software commonly used for statistical analysis in other methodological approaches. These include IBM SPSS (Field, 2024), JASP (Field et al., 2025), R (Field, 2025), SAS (Der & Everitt, 2008), and MATLAB (Martinez, 2011). To examine multiple sources of variation from the perspective of generalizability theory, in addition to these programs, specific tools have been developed for this purpose, such as SAGT (Hernández‑Mendo et al., 2016) and EduG (Cardinet et al., 2010).

## Quantitative parameters

The recorded data, an extremely rich qualitative component, are transformed into quantitative parameters that can be analyzed using a wide range of quantitative techniques, producing a set of quantitative results that are interpreted qualitatively. Therefore, OM does not rely on the complementarity of QUAL‑QUANT but on the integration of both components. Additionally, the quantification performed in OM is notably robust, because it not only considers frequency but also other primary parameters that offer researchers the possibility of mapping different components of spontaneous behavior (Anguera et al., 2017).

**Fig. 4:**
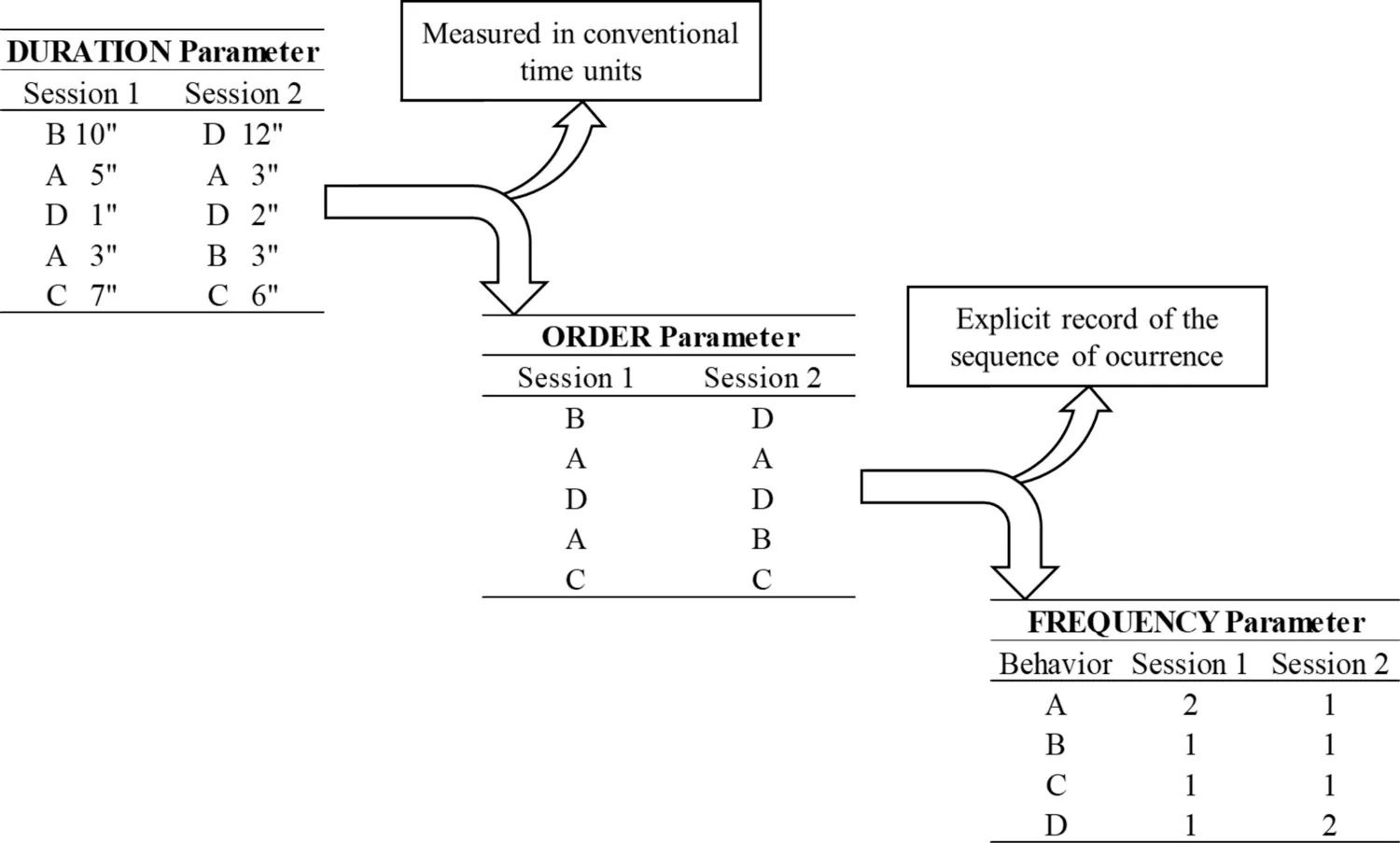
Information-complexity hierarchy of quantitative parameters

The quantitative parameters into which qualitative data can be transformed include the following: a) frequency, which captures how many times the observable phenomenon occurs; b) order, which captures the sequence in which successive occurrences take place; and c) duration, which captures how long successive occurrences last over time measured in conventional units (seconds, minutes, frames, etc.). Although frequency is the least informative parameter, it has historically been the most widely used. Conversely, the order and duration parameters can be reduced to less informative parameters. Thus, the order parameter can be stripped of sequence information and treated as a frequency parameter. Similarly, the duration parameter can be stripped of its time‑unit information and treated as an order parameter or stripped of everything except the count of occurrences and treated as a frequency parameter. However, the reverse process—moving up the hierarchy of informational power—is not possible (Anguera, 2003). Figure 4 presents the information-complexity hierarchy of quantitative parameters.

The specific quantitative parameter obtained depends on the type of qualitative data recorded. Any type of data (events or states, sequential or concurrent) can be transformed into frequency and order parameters. However, only sequential and concurrent states, that is, those recorded over time, can be transformed into duration parameters (Anguera et al., 2020). Table 2 shows the correspondence between observational design, qualitative data, and parameters.

**Table 2:** Correspondence between observational design, qualitative data, and parameters

Observational design	Qualitative data	Parameters
I or N/P or F/U	Type I: Sequential events	Frequency and Order
I or N/P or F/M	Type II: Concurrent events	Frequency and Order
I or N/P or F/U	Type III: Sequential states	Frequency, Order and Duration
I or N/P or F/M	Type IV: Concurrent states	Frequency, Order and Duration

*I* Idiographic; *N* Nomothetic; *P* Punctual; *F* Follow-up; *U* Unidimensional; *M* Multidimensional

## Data analysis in observational methodology

Historically, the quantitative analysis of behaviors in studies based on OM was limited to the mere counting of frequency. Consequently, analyses have relied, and still largely rely today, on conventional techniques such as descriptive and inferential statistics (Anguera et al., 2020; Blanco-Villaseñor et al., 2003). The observational design, like the observational instrument, the qualitative data recorded, and the resulting quantitative parameters, also determines which data-analytic techniques are used. This is based on the inherent characteristics of each quadrant, particularly the criteria for units of analysis and temporality, which determine which techniques can be applied (Anguera et al., 2001).

Quadrant I, idiographic follow-up designs, focuses on a single participant or on multiple participants who form a functional unit, and are recorded in multiple observation sessions. The set of techniques that can be employed in Quadrant I includes descriptive analyses, nonparametric tests, chi-square tests, ordinal, intraclass, and multiple correlation analyses, analysis of variance for continuous and categorical data, multivariate analysis of variance, linear and logistic regression analyses, multidimensional scaling, longitudinal analysis, trend analysis, and time-series analysis. Quadrant II, idiographic punctual designs, is the least robust from an analytical perspective, as they entail the collection of data from a single participant or functional unit at a single point in time. The set of techniques that can be employed in Quadrant II includes descriptive analyses, nonparametric tests, chi-square tests, ordinal and intraclass correlation analyses, logistic regression analyses, log-linear analyses, longitudinal analyses, and time-series analyses.

Quadrant III, nomothetic punctual designs, describes the distribution of a group of participants at a single point in time. This information is recorded in contingency tables containing categorical data, from which relationships and their strength can be examined. The techniques that can be used in Quadrant III include descriptive analyses, nonparametric tests, chi-square tests, ordinal, intraclass, and linear correlation analyses, logistic regression analyses, and log-linear analyses.

Quadrant IV, nomothetic follow-up designs, is the most comprehensive and analytically powerful. These designs allow concurrent study of the sequential progression of each group member or a series of punctual analyses that together form a quasi-follow-up over time (Anguera et al., 2018a, b). The techniques that can be used in Quadrant IV include descriptive analyses, nonparametric tests, chi-square tests, ordinal, intraclass, and multiple correlation analyses, analysis of variance for continuous and categorical data, multivariate analysis of variance, logistic regression analysis, log-linear analysis, multidimensional scaling, longitudinal analysis, trend analysis, and time-series analysis (Anguera & Hernández-Mendo, 2015; Anguera et al., 2001, 2018a, b; Blanco-Villaseñor et al., 2003).

In addition to these classical statistical procedures, OM has increasingly incorporated decision-tree techniques for predictive analysis in machine learning and applied statistics, such as Chi-squared Automated Interaction Detection (CHAID) and Classification and Regression Trees (CRT), for modeling non-linear relationships and nonparametric interactions (Lin & Fan, 2019). These techniques have recently demonstrated efficacy in a variety of concurrent variables and hierarchical decision structures commonly addressed using OM (e.g., Ilarri et al., 2025; Miranda et al., 2025; Sastre et al., 2022). Table 3 presents the analysis techniques by observational quadrant.

**Table 3:** Analysis techniques by observational quadrant

Analysis technique	Description	Applicability by quadrants
Descriptive analysis	Summarizes data by calculating frequencies and measures of central tendency, dispersion, or position (Kaur et al., 2018)	Quadrants I, II, III, and IV
Nonparametric tests	Test hypotheses without requiring assumptions about population parameters (Anguera & Hernández-Mendo, 2015)	Quadrants I, II, III and IV
Chi- square test	Assesses whether there is a statistically significant association between two categorical variables and evaluates the strength of this association (Anguera & Hernández-Mendo, 2015)	Quadrants I, II, III and IV
Linear correlation analysis	Examines the relationship or dependence between two continuous variables (Anguera & Hernández-Mendo, 2015)	Quadrant III
Ordinal correlation analysis	Examines the relationship or dependence between two ordinal variables (Anguera & Hernández-Mendo, 2015)	Quadrants I, II, III and IV
Intraclass correlation analysis	Assesses the consistency or agreement between repeated measurements of the same variable (Field, 2005)	Quadrants I, II, III and IV
Multiple correlation analysis​	Examines the relationship between one variable and a vector of two or more predictor variables, considering all possible linear combinations of those predictors (Krishnamoorthy & Xia, 2008)	Quadrants I and IV
Analysis of variance for continuous data	Partitions variability among groups on a continuous variable to estimate the influence of different sources of variation on a criterion variable (Anguera & Hernández-Mendo, 2015)	Quadrants I and IV
Analysis of variance for categorical data	Partitions variability in categorical outcomes to estimate the influence of various sources of variation (Anguera & Hernández-Mendo, 2015)	Quadrants I and IV
Multivariate analysis of variance	Partitions variability among groups on multiple continuous variables to estimate the influence of different sources of variation on two or more criteria (Field, 2024)	Quadrants I and IV
Linear regression analysis​	Models the relationship between predictor variables and continuous criterion variables (Anguera & Hernández-Mendo, 2015)	Quadrant I
Logistic regression analysis​	Models the relationship between predictor variables and dichotomous criterion variables (Anguera & Hernández-Mendo, 2015)	Quadrants I, II, III, and IV
Log-linear analysis	Examines relationships among multiple categorical variables to assess the fit of alternative models to the data (Anguera & Hernández-Mendo, 2015)	Quadrants II, III, and IV
Multidimensional scaling	Represents the structure of stimuli in a spatial configuration based on proximity or distance derived from their intercorrelations (Davison & Sireci, 2000)	Quadrants I and IV
Longitudinal analysis	Analyzes both interindividual and intraindividual change over time, and the interaction between these sources of variation (Hsiao, 1995)	Quadrants I, III, and IV
Trend analysis​	Identifies systematic patterns in data over time or as a function of an independent variable (Howell, 2018)	Quadrants I and IV
Time-series analysis	Analyzes patterns and dependencies in chronologically ordered data collected at regular time intervals (Anguera & Hernández-Mendo, 2015)	Quadrants I, II, and IV
Decision trees	Analyzes data segmented into conditional structures, profiles, and classification patterns by modeling nonlinear relationships and non-parametric interactions (Lin & Fan, 2019)	Quadrants I, II, III, and IV

Robust analyses based on frequency parameters can identify associations between categorical dimensions, analyze variability, or test causal relationships between dichotomous dimensions. However, transforming code matrices into order or duration parameters makes it possible to carry out additional, equally robust quantitative analyses of great importance (Anguera et al., 2020). Building on the inferential techniques described above, three techniques have been developed in recent decades that are considered endemic to OM because of their suitability for systematic records obtained in low‑intervention contexts: lag sequential analysis, polar coordinate analysis, and the detection of temporal patterns, commonly referred to as T‑patterns.

Lag sequential analysis detects behavioral patterns in interactions (Bakeman & Quera, 2011). These patterns can be analyzed prospectively (forward in the behavioral sequence) or retrospectively (backward) using positive or negative lags, respectively. For example, a behavior with a lag of + 2 occurs two positions after the behavior of interest in the behavioral sequence. Statistically significant categories at each lag before or after the behavior of interest (i.e., those whose conditional probability of occurring before or after the behavior of interest is higher than would be expected by chance) are extracted and become part of the behavioral pattern. This technique can be applied to order and duration parameters. When duration parameters are used, lag sequential analysis can be conducted with SDIS‑GSEQ or GSEQ5 (Bakeman & Quera, 2011). When order parameters are used, it can also be conducted with HOISAN (Anguera et al., 2018b).

**Fig. 5:**
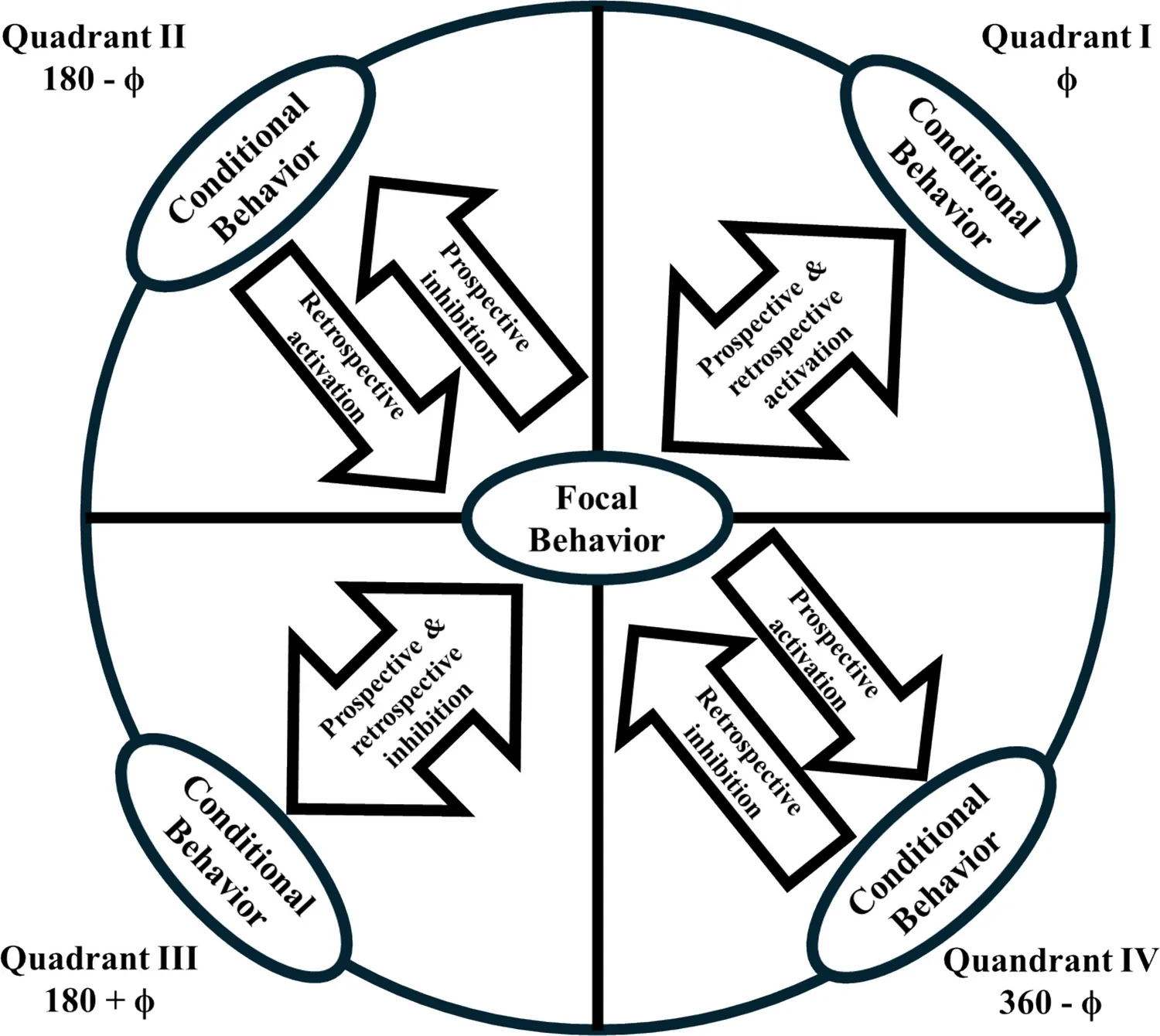
Relationship between focal and conditional behaviors as a function of quadrant

Polar coordinate analysis integrates the adjusted residuals from lag sequential analysis (typically using at least five prospective and five retrospective lags with respect to the focal behavior) and the Z_sum_ statistic to obtain prospective and retrospective Z_sum_ values that indicate activation or inhibition relationships (Sackett, 1980). This technique also applies to order and duration parameters and is implemented in HOISAN (Anguera et al., 2018b). A distinctive feature is the graphical representation of results as vectors on a coordinate axis, generating four quadrants. In quadrant I, the focal and conditional behaviors show a mutual activation pattern, such that the occurrence of one behavior increases the likelihood of the other. In quadrant II, the focal behavior prospectively inhibits the conditional behavior, which retrospectively activates the focal behavior. In quadrant III, the focal and conditional behaviors mutually inhibit each other. In quadrant IV, the focal behavior prospectively activates the conditional behavior, which retrospectively inhibits the focal behavior. The length of the vectors indicates the strength of the association (statistical significance) between focal and conditional behaviors (Sackett, 1980), and Fig. 5 displays these relationships by quadrant.

Time‑pattern detection is based on an algorithm that calculates temporal distances between behaviors and tests whether critical time intervals are more invariant than would be expected if behaviors were randomly and independently distributed over time. As a result, time patterns, or T‑patterns, are identified, helping to clarify how interactive transitions between behaviors unfold over time (Magnusson, 2016). This technique applies exclusively to duration parameters and is implemented in the Theme software (Magnusson, 2005; https://www.patternvision.com/).

Despite the differences among the three endemic analysis techniques in OM, they can all be applied to the same data matrix when a) the qualitative data recorded correspond to types III or IV (states measured in time units) and b) these data have been converted into duration parameters. This procedure provides complementary perspectives on the latent behavioral structures present in the data. Given OM’s history of relying on simple frequency counts or purely qualitative analyses (valuable approaches, but vulnerable to subjectivity), the capacity to triangulate results from three robust quantitative techniques—lag sequential analysis, polar coordinate analysis, and T‑pattern detection—represents a major advance in OM (Anguera et al., 2018b).

## Methodological quality in observational methodology

In scientific research, randomized controlled trials (RCTs) have long been regarded as the gold standard for methodological quality (MQ). As a result, most published MQ scales are based on this design. In fields where it is possible to manipulate and control variables, various strategies have been used to measure MQ empirically. One of the most common is the use of scales with construct validity and evidence of validity based on internal structure (Abad et al., 2011). These instruments typically consist of items grouped into dimensions that are summed to yield one or more global indices (Jefferson et al., 2009). Other widely used tools are guidelines and checklists, which resemble scales but generally lack extensive validation studies (Schulz et al., 2010).

In programs based on OM, design and implementation decisions place each study at a specific point on a continuum of MQ (Anguera, 2023). In the early years of the twenty-first century, research using OM grew markedly, prompting many scholars to focus on MQ in this mixed‑methods approach with the aim of promoting homogeneity and transparency both in the studies themselves and in the criteria used to evaluate their quality (Chacón-Moscoso et al., 2013).

Portell et al. (2015) proposed the Guidelines for Reporting Evaluations based on Observational Methodology (GREOM), which sets out 14 straightforward standards for conducting and reporting observational studies. Building on these guidelines, Chacón‑Moscoso et al. (2019) developed the Methodological Quality Checklist for Studies Based on Observational Methodology (MQCOM), a 16‑item Likert‑type checklist with evidence of content validity and inter‑rater reliability. Although instruments such as the Strengthening the Reporting of Observational Studies in Epidemiology (STROBE; von Elm et al., 2007) statement have improved the quality of observational studies more broadly, STROBE is not applicable to studies rooted in observational methodology.

Because OM is understood as a mixed‑methods approach (Anguera et al., 2012), several instruments can be used to assess MQ in this domain. Examples include the Methodological Rigor in Mixed Methods (Harrison et al., 2020), a checklist that specifies the components of mixed‑methods reports in sequence, and the Guidelines for Conducting and Reporting Mixed Research (Leech & Onwuegbuzie, 2010), which outline the formulation, design, and implementation of mixed‑methods studies through simple rules. In addition, the Mixed Methods Appraisal Tool (MMAT; Hong et al., 2018) assesses MQ not only in mixed‑methods studies but also in qualitative studies, descriptive quantitative studies, non‑randomized studies, and randomized controlled trials in health research. However, these instruments do not provide empirical evidence of their reliability or validity and instead focus on assessing the main dimensions of report quality.

In summary, the lack of MQ scales created substantial difficulties in the early years of the twenty-first century, despite the strong development of OM. Although there was broad consensus about the importance of MQ in OM studies, researchers and practitioners still struggled to design, implement, and evaluate their work. This, in turn, reduced transparency in reporting and increased the risk of overlooking potential biases (Portell et al., 2015). In academic settings, decision‑making bodies such as journal editorial boards also faced major challenges because they lacked solid empirical criteria to guide the selection and review of OM‑based manuscripts. As a result, the accumulation of high‑quality scientific knowledge grounded in OM was hindered (Chacón‑Moscoso et al., 2013). To address these challenges, MQ assessment in observational‑methodology studies has advanced considerably, as reflected in the growing use of systematic reviews to synthesize evidence from OM‑based research (Anguera, 2023). This line of methodological work began with the application of GREOM (Portell et al., 2015) to primary football studies using OM, examining the link between how research is reported and its underlying methods and revealing latent quality profiles in the existing literature (Preciado et al., 2019, 2021).

More recently, drawing on MQCOM (Chacón‑Moscoso et al., 2019), the Methodological Quality Scale for Studies Based on Observational Methodology (MQSOM) was developed (Sanduvete‑Chaves et al., 2025). This 11‑item scale assesses a second‑order dimension of global MQ through two first‑order dimensions: design quality and the quality of measurement and analysis. It specifies the minimum methodological requirements needed to improve the quality of OM‑based studies. MQSOM has shown adequate reliability and validity evidence based on its internal structure (Sanduvete‑Chaves et al., 2025), as well as convergent–discriminant validity (López‑Arenas et al., in press). The MQSOM coding manual is available at https://osf.io/wju3r/files/mvk52.

Building on earlier efforts to synthesize OM‑based evidence (Preciado et al., 2019, 2021), MQSOM has been applied to primary studies on soccer (López-Arenas et al., 2026a) and basketball (López-Arenas et al., 2026b). These systematic reviews identified methodological trends and clarified methodological consistency by combining MQSOM scores, dimension scores, and characteristics of the research procedures to derive MQ profiles ordered from lower to higher levels of quality. They also revealed a substantial body of work that uses OM but does not label it as such, instead referring to performance analysis (e.g., Rampinini et al., 2007; Young et al., 2023), notational analysis (e.g., James et al., 2002; Vencúrik et al., 2021), or time‑motion analysis (e.g., Di Salvo et al., 2007; Ferioli et al., 2023). In addition, many studies with ex post facto designs can be classified as OM research, including work based on direct observation (e.g., Sgrò et al., 2016; Towner et al., 2023) and indirect observation (e.g., Thomas et al., 2006; Pérez‑Chao et al., 2024). This methodological heterogeneity, together with the lack of sustained collaboration or consensus between quantitative and mixed‑methods perspectives, has limited the accumulation of high‑quality scientific knowledge grounded in OM.

Future initiatives therefore need to pursue a dual objective. First, they should promote the dissemination of MQSOM among researchers, practitioners, journal editorial boards, and international initiatives such as the EQUATOR Network. This dissemination will help consolidate systematic observation as a methodological option that is both rigorous and useful. Second, they should expand the synthesis of OM‑based evidence beyond sport contexts to other fields such as psychology and education. Doing so will enable a more comprehensive and systematic examination of different approaches to the study of spontaneous behavior and foster a more integrated understanding of their methodological rigor. In this regard, future syntheses should also incorporate related methodological descriptors such as Systematic Coding of Observed Human Behavior (SCOBe; Hillen et al., 2025) and research methods for studying daily life (Mehl & Conner, 2012) in their search strategies.

## Conclusion

The growing number of studies based on OM is encouraging for the coming decades. Strengthening a flexible yet rigorous OM through methodological innovations in both quality assessment and evidence synthesis will help consolidate this approach as essential for explaining behavior in everyday contexts. We hope this work will serve as an introduction for researchers interested in applying OM in their own fields, supporting their training, enriching the scientific record, and fostering a methodological culture grounded in systematic observation.

## Data Availability

Data sharing not applicable to this article as no datasets were generated or analyzed during the current study. MQSOM codification manual is available at https://osf.io/wju3r/files/mvk52.
